# A model of the impact of land use change on its carbon sequestration capacity

**DOI:** 10.1371/journal.pone.0323645

**Published:** 2025-05-29

**Authors:** Xu Huang, Wei Lyu, Haiyan Ye, Longshun Su

**Affiliations:** 1 Neijiang normal university, Neijiang City, China; 2 Sichuan Key Laboratory of Ecological Environment Data Analysis and Green Development Intelligent Decision-Making for the Upper Yangtze River, Neijiang Normal University, Neijiang City, China; 3 Yuxi Normal University, Yuxi City, China; 4 No.22 Middle School, Zigong City, China; 5 Yuxi Water Conservancy Construction Co., Ltd., Yuxi City, China; Central University of Rajasthan, INDIA

## Abstract

Different land-use types have different carbon sequestration capacities, and changes in land-use type directly cause changes in carbon sequestration capacity. To understand the change trend of carbon sequestration capacity, it is necessary to quantify the specific quantity of the impact of land use change on carbon sequestration capacity to indirectly estimate the change in carbon sequestration capacity by monitoring the change in land use type. Based on the analysis of the changing trends of land use type data and ecological carbon sequestration data in the research area, this study establishes an impact model (IM) of land use change on ecological carbon sequestration capacity, quantifying the specific quantity of the impact of land use change on ecological carbon sequestration capacity from two aspects: the impact of land use type transition in and the impact of land use type transition out. Through verification of the accuracy of the model estimation, it is expected that the impact of land use change on carbon sequestration capacity in Sichuan mountainous areas in 2025 and 2030 will be -20754 tons and -30837 tons, respectively, and the total ecological carbon sinks will be 132 and 133 million tons, respectively. Based on the trend analysis of the total carbon sequestration and the IM estimation data, the impact of land use change on ecological carbon sequestration capacity tends to stabilize (the impact will fluctuate around -31156 tons), and the trend of a significant increase in total carbon sequestration is not obvious. The IM quantifies the specific quantity of the influence of land-use type change on carbon sequestration capacity and can dynamically analyze the external factors that cause the change in carbon sequestration capacity, which is of great significance for monitoring the change law of carbon sequestration capacity.

## Introduction

With the proposal of carbon neutrality targets, research on vegetation carbon sequestration has gradually become a hot topic [1–3]. Many studies have been conducted on the estimation methods, models, trends, and impact of external conditions on ecological carbon sinks for carbon sequestration. Research on the impact of land use change on ecological carbon sink function has also achieved good results [4]. The machine learning model proposed by Mo et al. shows that human activities significantly reduce forest carbon storage compared to the natural potential, and optimizing land resource management can significantly enhance carbon sinks [5]. Sha et al. proposed that by optimizing land management measures, such as forest nurturing and grassland restoration, the global terrestrial ecosystem can add approximately 3.5–4 billion tons of carbon annually, equivalent to one-third of global fossil fuel emissions [6]. Studies have specifically pointed out that grasslands and farmland outside of forests have a higher potential for carbon sequestration, and the rational use of land has a greater impact on carbon sequestration capacity. Researchers have studied land-use conflicts in the study area from the perspective of ecological carbon sinks and identified land-use conflicts in the study area from the perspective of improving ecological carbon sink capacity [7–9]. Raj et al. evaluated the land use dynamics in the Aravalli Mountains of India using a combination of geographic space and CART methods, and predicted future land use changes, indirectly reflecting the impact of future land use changes on ecological carbon sequestration capacity [10]. Pekka et al. conducted a study on land use and carbon emissions in Finland and found that forests can effectively reduce carbon emissions, and strengthening forest management can slow down the occurrence of greenhouse effects [11]; Zhou et al. conducted a survey of 7800 plots nationwide to evaluate the current status and rate of carbon sequestration in China’s forest ecosystems, predict their carbon sequestration potential, and explore the mechanisms of carbon sequestration in forest ecosystems [12]; Wang et al. quantitatively evaluated the impact of land use change on carbon balance and found that returning farmland to forests can increase carbon dioxide absorption [13]; Liu et al. analyzed the correlation between land use change and ecological carbon sink based on remote sensing data from 2005 and 2015, and found that the reduction of ecological risks caused by land use change can be directly reflected in the improvement of ecological carbon sink capacity [14]; Chen et al. analyzed the spatiotemporal distribution characteristics between carbon sink values and climate regulation values on the Qinghai Tibet Plateau based on land use data, and found that the interannual variation of carbon sinks was very small [15]. Zeng et al. used the Carnegie Ames Stanford method (CASA) model to estimate ecological carbon sequestration in the study area and studied the ecological carbon sequestration capacity of vegetation under different land-use type [16]. Strengthening research on the impact of land use change on ecological carbon sinks can help understand the effects of land use change, ecological carbon sink capacity, and future trends, thereby contributing to achieving carbon neutrality goals.

Although there have been many studies on the correlation between land use and carbon sequestration capacity, current research has only revealed the correlation between the two and has not specifically quantified it [17,18]. As far as the current research status is concerned, there is a lack of calculation methods and models to calculate the impact of land-use type changes on carbon sequestration capacity, and the specific impact of land use changes on carbon sequestration capacity cannot be clarified. Therefore, relevant models are needed to quantify the impact of land use change on carbon sequestration capacity.

How can we quantify the impact of land use change on ecological carbon sequestration capacity? This study focused on the mountainous areas of Sichuan Province, which have strong carbon sequestration capabilities, as the research area. Based on the analysis of the changes in land use and carbon sequestration capacity in the Sichuan mountainous areas, the IM was established from two aspects: the impact of land use type transition in and the impact of land use type transition out. The model was used to quantify the impact of land-use type changes on carbon sequestration capacity to more accurately grasp the basic situation of carbon sequestration capacity in the study area.

## Study area, data and methods

### Study area

Considering the abundant ecological resources and frequent changes in land-use type in Sichuan mountainous areas, this study chose Sichuan mountainous areas as the research area. However, classification methods for mountainous areas are diverse, and a unified classification standard has not yet been established. Based on the literature, the selection of mountainous area definition indicators is consistent, and mountainous areas are qualitatively and quantitatively described by factors such as absolute height, relative height, and slope [19]. According to the results of Zhang et al., the following definition criteria for Sichuan mountainous areas were selected [20,21]:

1. The area with elevation less than 500 m, the terrain fluctuation is greater than 50 m;
2. The elevation between 500m-2500m area, terrain ups and downs higher than 100m or slope greater than 25 °area;
3. Area with elevations above 2500 m.

**Fig 1:**
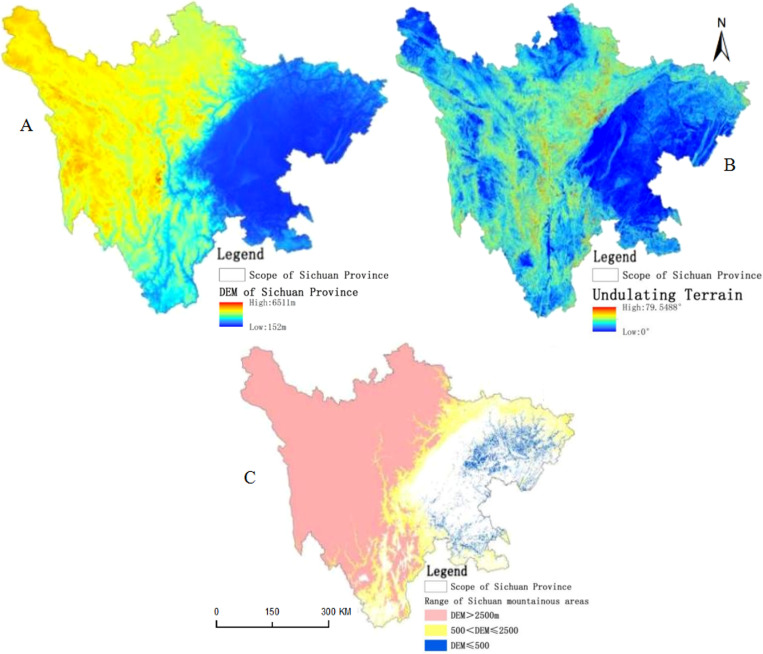
Schematic diagram of Sichuan mountainous areas acquisition (DEM Dataset is provided by National Ecosystem Science Data Center, National Science & Technology Infrastructure of China (http://www.nesdc.org.cn).

Using ArcGIS software and Sichuan ASTER DEM data (spatial resolution 30m, Fig 1A) [22], the DEM dataset was provided by the National Ecosystem Science Data Center and the National Science and Technology Infrastructure (http://www.nesdc.org.cn). The terrain undulation data (Fig 1B) was obtained through DEM calculations. Generate range data for Sichuan mountainous areas based on the above standards (Fig 1C).

### Data collection, processing, and trend analysis

#### Land use data.

**Fig 2:**
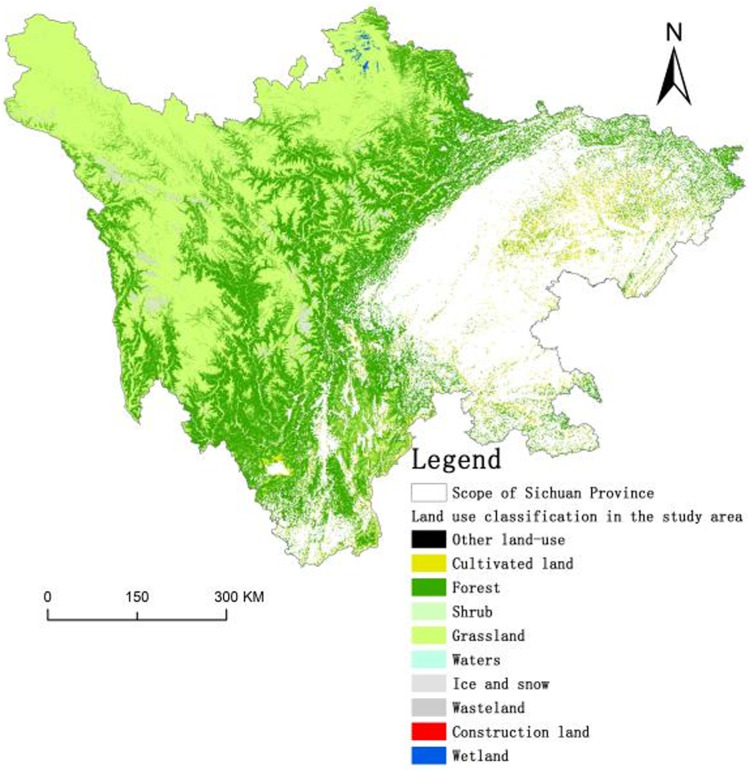
Distribution of land use status in Sichuan mountainous areas in 2020 (DEM Dataset is provided by National Ecosystem Science Data Center, National Science & Technology Infrastructure of China (http://www.nesdc.org.cn).

The data used in this study were mainly based on Landsat TM remote sensing images from 2000 to 2020 as data sources. Erdas Imagine, ENVI, and ArcGIS were used to preprocess remote sensing images from different periods in Sichuan mountainous areas. Supervised classification and human-computer interaction methods are used to interpret and test the accuracy. Combined with Google Earth software to correct the interpretation results, the vector data of land use in Sichuan in different years are obtained and the area of land types are counted. The total accuracy of image interpretation obtained by the accuracy test was higher than 83%, and the kappa coefficient was higher than 0.81. The accuracy of the land use data meets the research requirements [22–24]. The land-use type in Sichuan mountainous areas include construction land, forest, grassland, cultivated land, shrub, lake, wetland, wasteland, ice, and snow, with a spatial resolution of 30 m. The land-use distribution map for the research area in 2020 is shown in Fig 2.

#### Ecological carbon sequestration data.

**Fig 3:**
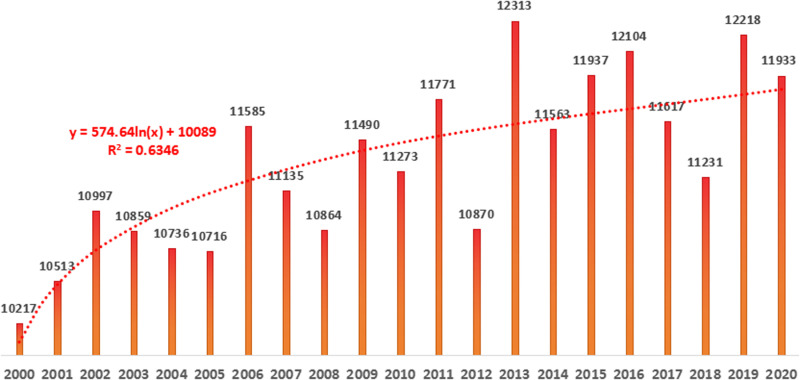
The total amount of ecological carbon sequestration from 2000 to 2020.

Different carbon sequestration estimation models are suitable for estimating carbon sequestration in areas with different topographic features. Based on the small- and medium-scale characteristics of Sichuan mountainous areas, accuracy of the model estimation, and difficulty of data acquisition, the CASA model was selected to estimate the total amount of ecological carbon sequestration in Sichuan mountainous areas. The CASA model not only has the advantages of simple structure, high reliability, easy access to parameters, and relatively high accuracy of estimation results, but it is also widely used in the estimation of vegetation NPP at medium and large spatial scales [25]. However, according to existing research, the CASA model assigns a unified value of maximum light energy utilization efficiency (e = 0.389) without considering the influence of vegetation types, terrain factors, and other factors on the estimation results. Based on these shortcomings, Huang et al. improved the model and used the improved CASA model to estimate the total ecological carbon sequestration in Sichuan mountainous areas (Fig 3) [26]. The total ecological carbon sequestration showed an increasing trend (R^2^ = 0.6346). As the carbon sequestration area of the ecosystem remains unchanged, it can be seen that the carbon density in Sichuan mountainous areas generally shows an increasing trend, but the increase gradually slows.

**Fig 4:**
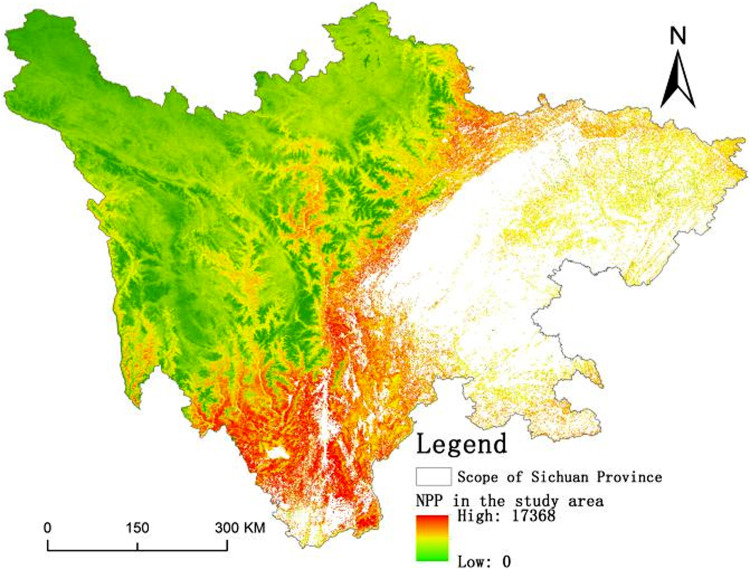
The distribution map of ecological carbon sequestration in Sichuan mountainous areas in 2020 (DEM Dataset is provided by National Ecosystem Science Data Center, National Science & Technology Infrastructure of China (http://www.nesdc.org.cn).

Combined with the spatial distribution of total ecological carbon sequestration (Fig 4) and land-use status data (Fig 2) in the Sichuan mountainous areas, the main types of utilization in the high-value areas of carbon sequestration in the mountainous areas are forests and shrubs, which are mainly concentrated in the mountainous altitude transition zone. The carbon sequestration capacity of the low-altitude area in mountainous areas in general and the main land use type is cultivated land. In high-altitude mountainous areas, the ecological carbon sequestration capacity is weak, and the main land-use type is grassland. Overall, the regional change in carbon sequestration capacity is not large, and the interannual change in total carbon sequestration mainly comes from the low-altitude area of mountainous areas, the transition zone between low altitude and high altitude. This is mainly because the ecological carbon sequestration capacity is affected by external factors, particularly the influence of temperature and precipitation.

#### Trend analysis of data changes.

**Fig 5:**
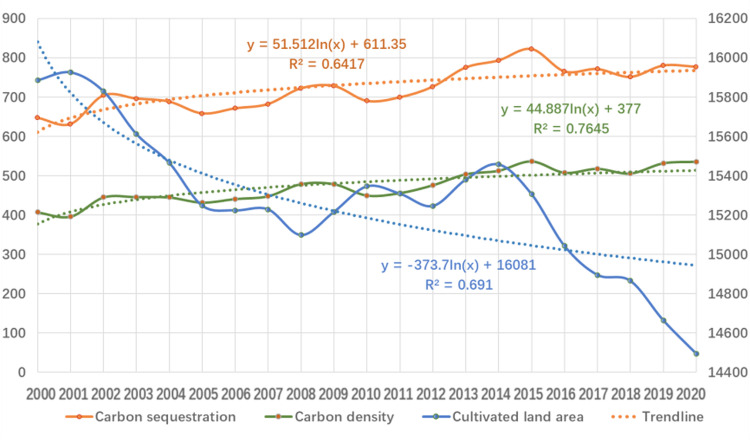
Trends of cultivated land area, carbon sequestration and carbon density from 2000 to 2020.

**Fig 6:**
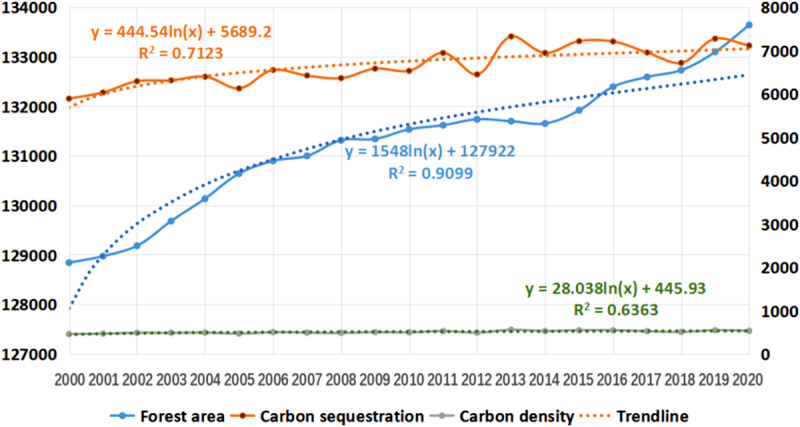
Trends of forest area, carbon sequestration and carbon density from 2010 to 2020.

**Fig 7:**
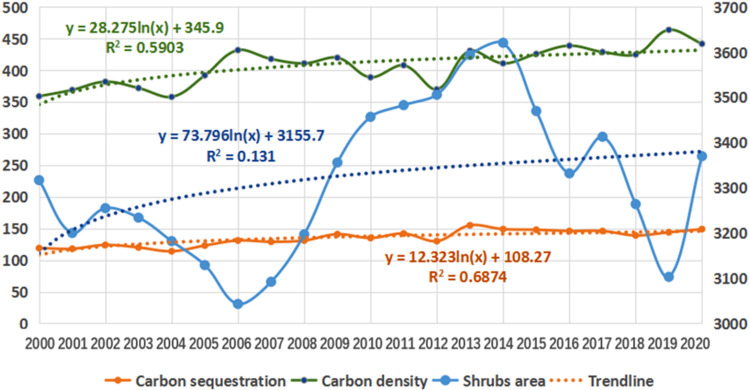
Trends of shrub area, carbon sequestration and carbon density from 2010 to 2020.

**Fig 8:**
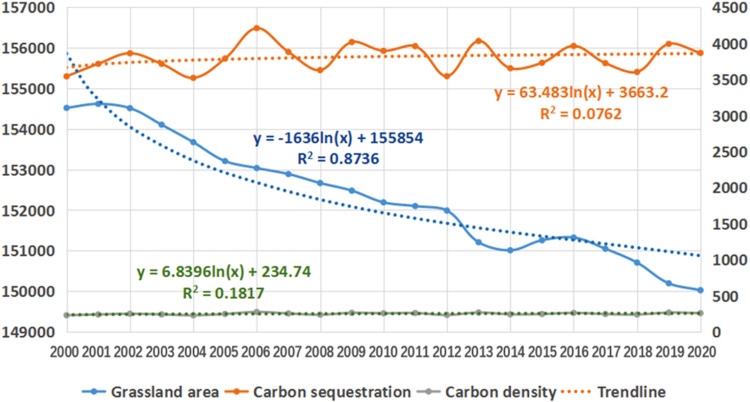
Trends of grassland area, carbon sequestration and carbon density from 2010 to 2020.

**Fig 9:**
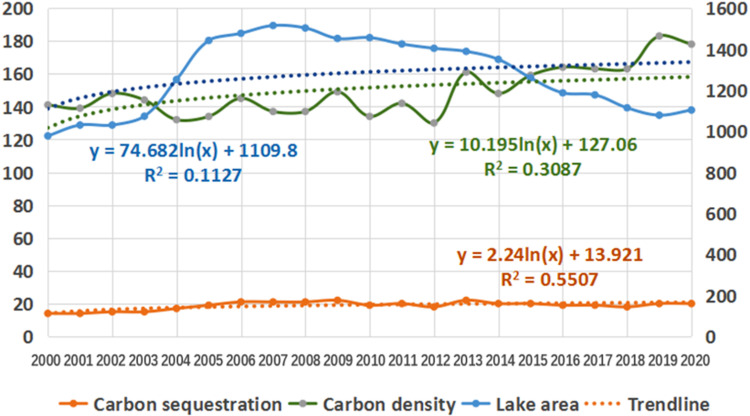
Trends of lake area, carbon sequestration and carbon density from 2010 to 2020.

**Fig 10:**
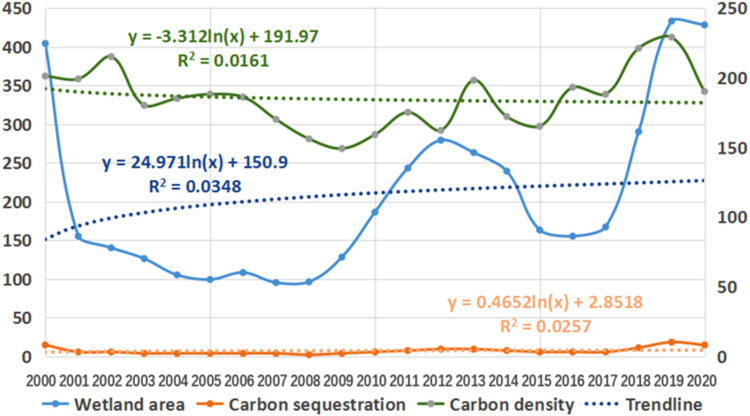
Trends of wetland area, carbon density and carbon sequestration from 2010 to 2020.

**Fig 11:**
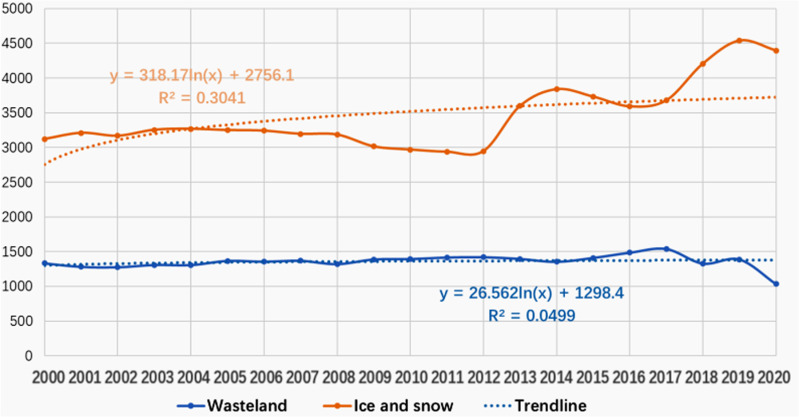
Trend of Ice and Snow, and Wasteland Area from 2010 to 2020.

Based on the basis of obtaining the above data, the ecological carbon sequestration data and land use data of the research area from 2000 to 2020 were used to calculate the carbon density data of different land-use type and analyze their changes over time. These three types of data and their changing patterns serve as the basic data for estimating the IM. The basic data are listed in Table 1, and the variation pattern of the basic data is shown in Figs 5–11.

**Table 1:** Basic data statistics table from 2000 to 2020 (Units: km^2^,Ton, g/m^2^).

Year	Construction land	Cultivated land	Carbon sequestration	Carbon density	Forest	Carbon sequestration	Carbon density	Shrub	Carbon sequestration	Carbon density
2000	107	15887	647	407	128841	5889	457	3317	119	359
2001	108	15927	631	396	128973	6033	468	3200	118	369
2002	110	15831	704	445	129183	6291	487	3255	124	382
2003	111	15613	696	446	129681	6312	487	3234	120	372
2004	115	15466	688	445	130134	6396	492	3182	114	358
2005	119	15248	658	432	130640	6127	469	3129	123	392
2006	120	15224	671	441	130897	6551	501	3043	131	432
2007	122	15229	682	448	130998	6423	490	3092	129	418
**Year**	**Grass land**	**Carbon sequestration**	**Carbon density**	**Lake**	**Carbon sequestration**	**Carbon density**	**Wetland**	**Carbon sequestration**	**Carbon density**	**Total carbon sequestration**
2008	123	15098	722	479	131314	6362	485	3197	131	411
2009	126	15216	728	479	131341	6582	501	3356	141	420
2010	131	15346	690	450	131534	6532	497	3457	135	389
2011	133	15309	699	456	131617	6946	528	3483	142	408
2012	135	15246	726	476	131737	6449	490	3506	130	370
2013	138	15380	775	504	131698	7325	556	3594	155	431
2014	142	15458	793	513	131652	6946	528	3621	149	411
2015	148	15307	822	537	131918	7216	547	3470	148	426
2016	154	15043	765	508	132393	7209	545	3332	146	439
2017	158	14895	771	518	132591	6954	525	3413	146	429
2018	162	14865	751	506	132728	6715	506	3264	139	425
2019	166	14664	780	532	133097	7273	546	3103	144	464
2020	171	14494	777	536	133641	7115	532	3370	149	442
**Year**	**Grass land**	**Carbon sequestration**	**Carbon density**	**Lake**	**Carbon sequestration**	**Carbon density**	**Wetland**	**Carbon sequestration**	**Carbon density**	**Total carbon sequestration**
2000	154516	3540	229	977	14	141	404	8	201	10217
2001	154615	3714	240	1030	14	139	155	3	199	10513
2002	154509	3860	250	1030	15	148	140	3	215	10997
2003	154101	3713	241	1073	15	144	126	2	180	10859
2004	153671	3519	229	1252	17	132	105	2	185	10736
2005	153206	3787	247	1442	19	134	99	2	188	10716
2006	153034	4208	275	1477	21	145	108	2	186	11585
2007	152886	3879	254	1515	21	137	95	2	170	11135
2008	152665	3626	237	1503	21	137	96	1	156	10864
2009	152478	4016	263	1452	22	149	128	2	149	11490
2010	152189	3894	256	1456	19	134	186	3	159	11273
2011	152097	3959	260	1425	20	142	243	4	175	11771
2012	151988	3543	233	1404	18	130	279	5	162	10870
2013	151206	4030	267	1389	22	161	263	5	198	12313
2014	151008	3652	242	1350	20	148	239	4	172	11563
2015	151255	3728	246	1260	20	159	163	3	165	11937
2016	151321	3961	262	1187	19	164	155	3	193	12104
2017	151046	3723	246	1177	19	163	167	3	188	11617
2018	150704	3601	239	1114	18	163	290	6	221	11231
2019	150194	3990	266	1078	20	183	433	10	229	12218
2020	150024	3865	258	1103	20	178	428	8	190	11933

Combined with Table 1 and Fig 5, the area of cultivated land in mountainous areas shows a trend of stage reduction, whereas carbon sequestration and carbon density show an increasing trend, and the increasing trend is more consistent. The carbon sequestration and carbon density of cultivated land did not decrease with a decrease in area, and the average annual growth of carbon density was 7 g/m^2^.

As shown in Table 1 and Fig 6, the forest area in Sichuan mountainous area is increasing, the growth of forest carbon sequestration and carbon density is relatively gentle, and the average annual growth of carbon density is 5 g/m^2^.

Combined with Table 1 and Fig 7, the shrub area in the Sichuan mountainous areas fluctuates greatly, but the carbon sequestration and carbon sequestration density of shrubs show a stable increasing trend. The carbon density of shrubs does not change with the change of area, and the carbon density increases by 5.3 g/ m^2^ annually.

Combined with Table 1 and Fig 8, the grassland area in Sichuan mountainous area decreased year by year, and the carbon sequestration and carbon density of grassland increased with the decrease in grassland area, and the change was relatively stable. The carbon density of grasslands showed a slow growth trend over time, with an average annual growth of 1.9 g/m^2^.

Combined with Table 1 and Fig 9, the lake area in Sichuan mountainous areas changes greatly, which is mainly affected by precipitation factors, but it is basically stable in general, and the trend line is basically the same as the horizontal line. The carbon sequestration capacity of the lake does not change with the change of area, and its carbon sequestration capacity is relatively stable. The carbon density of the lake increased slowly, with an average annual increase of 2.65 g/m^2^.

Combined with Table 1 and Fig 10, the area of the mountain wetland and the total amount of carbon sequestration fluctuate greatly between years and are mainly affected by climate, but the change in carbon density is relatively stable, and the trend line is basically the same as the horizontal line.

Combined with Table 1 and Fig 11, the areas of ice and snow in the Sichuan mountainous areas show a slow growth trend, while the area of wasteland changes more smoothly, and the trend line is consistent with the horizontal line.

### Establishment, estimation, and verification of IM

#### Establishment of the IM.

To accurately grasp the impact of land use change on ecological carbon sequestration, the paper constructed and used to estimate the specific impact of land use change on ecological carbon sequestration. The equations for the model are as follows:


$$GTYX=\sum {\mathrm{\backslash nolimits}}_{n}\left(ZR_{i}+ZC_{i}\right)$$ (1)

$$ZR_{i}=\sum {\mathrm{\backslash nolimits}}_{n}\left(\sum_{i}^{t}\left(S_{t}*(\rho_{i}*(1+\beta_{i})-\rho_{t}*(1+\beta_{t}))*a_{\left(t,i\right)}\right)\right),i=1,2,..,n,t\leq n$$ (2)

$$ZC_{i}=\sum {\mathrm{\backslash nolimits}}_{n}\left(\sum_{i}^{t}\left(\left(S_{i}*(\rho_{t}*(1+\beta_{t})-\rho_{i}*(1+\beta_{i}))*a_{\left(i,t\right)}\right)\right)\right),i=1,2,...,n,t\leq n$$ (3)

In the equation 1–3, GTYX is the total impact of land use on ecological carbon sequestration, ZR_i_ is the impact of the transfer of other land-use type to the i-th land use type, ZC_i_ is the impact of the transfer of the i-th land use type to other land-use type, S_t_ is the area of the t-th land use type, S_i_ is the area of the i-th land use type, n is the number of land-use type, *a*_(t, i)_ is the transfer probability of the t-th land use type to the i-th land use type, *a*_(i, t)_ is the transfer probability of the i-th land use type to the t-th land use type. ρ_t_ and ρ_i_ are the carbon density of the t-th and i-th current land use type, and *β*_t_ and *β*_i_ are the increase and decrease coefficients of the carbon density of the t-th and i-th land use type.

The IM divides the impact of land-use change on ecological carbon sequestration capacity into the impact of land-use types, namely, transfer-in (ZR) and transfer-out (ZC). The transfer of unfavorable carbon sequestration was negative (from higher carbon density to lower carbon density), and the transfer of strong carbon sequestration was positive (from lower carbon density to higher carbon density). The sum of the transfer values of the nine land-use types can determine the impact of land-use change on carbon sequestration.

**Fig 12:**
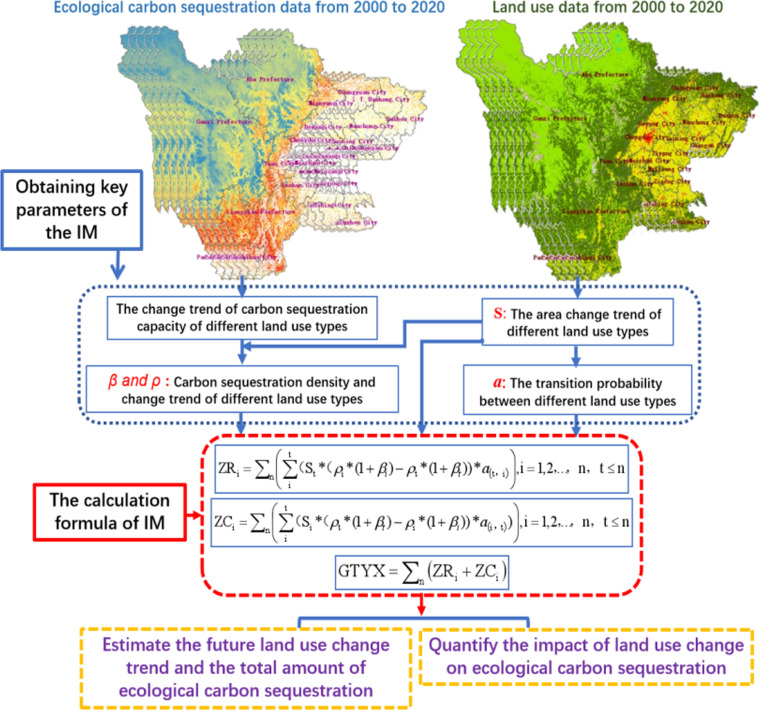
Data processing flowchart of the IM (DEM Dataset is provided by National Ecosystem Science Data Center, National Science & Technology Infrastructure of China (http://www.nesdc.org.cn).

The specific data-processing process of the IM is shown (Fig 12).

1. Land use data and ecological carbon sequestration datasets from 2000 to 2020 were collected in the research area, along with the changes in land use type area (S), carbon sequestration amount, and carbon sequestration density (*β*).
2. Combining land use data and ecological carbon sequestration data, analyze the trend (*ρ*) of carbon sequestration density changes in different land-use type from 2000 to 2020, and calculate the transition probability between different land-use type (*a*)
3. Using *β*, *ρ*, *a*, and S obtained in the above steps as four important parameters to calculate the transfer-in (ZR) and transfer-out (ZC) of different land-use type in the IM. By integrating the impact of different land-use types on ecological carbon sequestration, the total impact of land-use changes on ecological carbon sequestration capacity (GTYX in Fig 12) in the study area was obtained, and the future trend of changes in land use and ecological carbon sequestration was estimated.

#### Estimation and verification of the IM.

From Equations 1–3, it can be seen that the unknown main parameters in the model include land use transition probability *a*, land use carbon density increase/decrease coefficient *β*, and land use-type area S. Before conducting the estimation in the IM, it is necessary to use existing land use data and carbon sequestration data to estimate *a*, *β*, and S.

**Calculation of transition probability (a):** Different land-use types have different ecological carbon sequestration capabilities, and the transition probability between different land types is a key parameter for estimating the impact of input and output in the IM. This study used the established land use type transfer matrix equation (Equation 4) and land use data to calculate the average value of land use transfer probability from 2000 to 2020 (Table 4), which serves as the transfer probability between different land-use type in Sichuan mountainous areas in the future.

**Table 2:** Statistics of average land use transition probability a from 2000 to 2020.

	Cultivated land	Forest	Shrub	Grass land	Lake	Ice and snow	Wasteland	Construction land	Wetland
Cultivated land	0.8611	0.1008	0.0052	0.0294	0.0019	0.0000	0.0000	0.0016	0.0000
Forest	0.0090	0.9852	0.0056	0.0001	0.0000	0.0000	0.0000	0.0001	0.0000
Shrub	0.0131	0.1493	0.6479	0.1761	0.0000	0.0000	0.0000	0.0000	0.0000
Grass land	0.0029	0.0061	0.0024	0.9802	0.0005	0.0000	0.0069	0.0000	0.0011
Lake	0.0030	0.0031	0.0000	0.1299	0.7681	0.0002	0.0893	0.0008	0.0000
Ice and snow	0.0000	0.0000	0.0000	0.0196	0.0070	0.6892	0.2821	0.0000	0.0000
Wasteland	0.0000	0.0004	0.0000	0.1709	0.0036	0.0795	0.7443	0.0000	0.0000
Construction land	0.0000	0.0000	0.0000	0.0001	0.0633	0.0000	0.0000	0.9366	0.0000
Wetland	0.0002	0.0004	0.0001	0.2738	0.0053	0.0000	0.0000	0.0000	0.6969

**Table 3:** Statistics of predicted value of grey model of land-use type areas (Unit:km^2^).

Year	Construction land	Cultivated land	Forest	Shrub	Grass land	Wetland
2025	198.27	14179.34	134407.22	3074.83	149164.50	436.88
2030	229.55	13734.98	135526.69	2910.03	148164.52	537.72

**Table 4:** Statistics of predicted values of carbon density grey prediction model (Unit:gC/m^2^).

Year	Carbon density of cultivated land	Forest carbon density	Shrub carbon density	Grassland carbon density	Lake carbon density	Wetland carbon density
2025	574.6	540.4	485	257.46	205.08	237.26
2030	612.91	545.85	520.47	260.46	231.92	264.97


$$a_{i}=\frac{\left(100*M_{\left(i,t\right)}+M_{\left(q,t+n\right)}\right)*Count\left(\left(100*M_{\left(i,t\right)}+M_{\left(q,t+n\right)}\right)\right)*k}{S_{\left(i,t\right)}}$$ (4)

In the equation, *a*_i_ is the transition probability of the i-th land-use type; *M*_(i, t)_ is the i-th land-use type number in t time period, and *M*_(q, t)_ is the q-th land-use type number in t + 1 time period. The utilization types are divided into cultivated land-1, forest-2, shrub-3, grassland-4, water body-5, ice and snow-6, wasteland-7, construction land-8, wetland-9, generally i = q; *n* is the time interval, and the unit is year; *k* is the grid area of the raster data; count () is the counting function; S _(i, t)_ is the total area of the i-th utilization types in t time.

**Fig 13:**
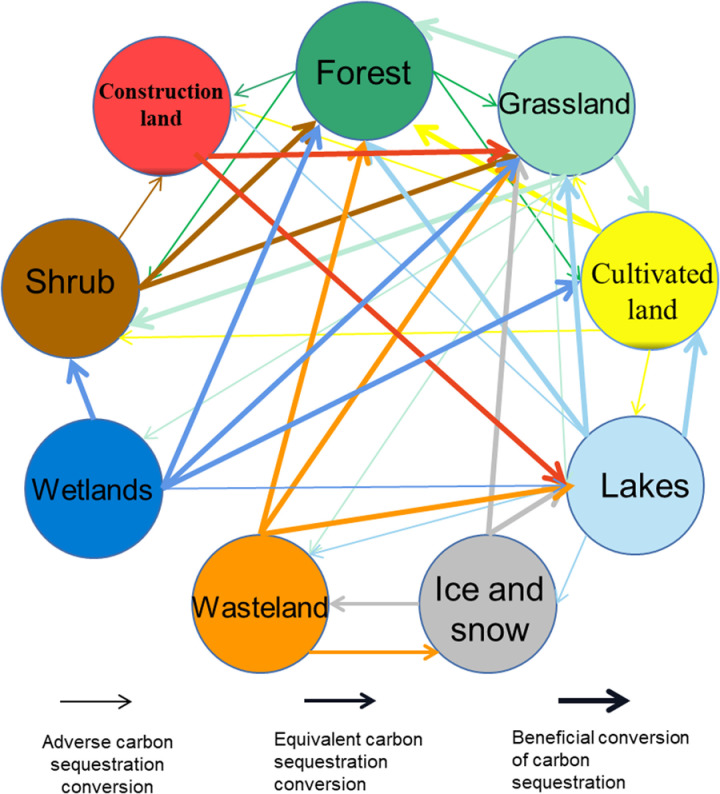
Spherical diagram of different land-use type conversion directions.

To reflect the mutual transfer relationship between land-use type in Sichuan mountainous areas more intuitively, the transfer ball of land-use type is drawn according to the transfer probability in Table 2 (Fig 13). In the Fig, most types of outward transfer are grassland and lakes, and the least is construction land. The most inward transfer type was grassland and the least was wetland. There are 47 transfer routes of land-use types in Sichuan mountainous areas, of which 21 are conducive to the transfer of carbon sequestration, 24 are not conducive to the transfer of carbon sequestration, and 2 are the transfer between the same ability of carbon sequestration.

**β and S estimation:** To estimate the change in land use type (S) and carbon density (***β***) in Sichuan mountainous areas, the Grey Prediction Model and ARIMA prediction model in the most commonly used time series prediction model were selected to predict the area and carbon density of different land-use type. The Grey Prediction model is suitable for small samples, obvious trends, and medium-to long-term predictions, and has the advantage of being simple and efficient. The ARIMA prediction model is suitable for data sufficiency, complex temporal patterns, and short-term high-precision predictions, with the advantages of flexibility and statistical rigor. In practical applications, the two models are usually combined and used interchangeably to balance the prediction needs in different scenarios.

***The grey prediction model:*** The Grey Prediction Model is a time-series prediction method for predicting related systems with important uncertain parameters. The degree of difference in the development trend between the factors of the grey prediction identification system, that is, a detailed analysis of the similarity, generation, and processing of the original data, to find the general law of the establishment and change of the system, calculate the data sequence with strong regularity, and then establish the corresponding differential equation model to predict the future development trend. The grey prediction model is constructed using a series of quantitative values that reflect the characteristics of the predicted object observed at equal time intervals to predict the characteristic quantity at a certain time in the future or the time to reach a certain characteristic quantity [27,28]. The land use and carbon sequestration data of Sichuan mountainous areas from 2000 to 2020 were imported into the grey prediction model to estimate the area and carbon density data of land-use type for 2025 and 2030. The results are as follows (Table 3):

***ARIMA prediction model:*** The basic idea of the ARIMA model is to regard the data sequence formed by the predicted object over time as a random sequence and use a mathematical model to describe this sequence. Once the model is identified, it can predict the future value from past and current values of the time series [29,30]. The land use and carbon sequestration data of Sichuan mountainous areas from 2000 to 2020 were imported into the ARIMA prediction model to estimate the area and carbon density data of land-use types for 2025 and 2030.. The results are as follows (Tables 5 and 6):

**Table 5:** Statistics of predicted value of ARIMA model of land-use type areas (Unit: km^2^).

Year	Construction land	Cultivated land	Forest	Shrub	Grass land	Wetland
2025	208.34	16507.03	150877.57	3853.53	171672.30	279.04
2030	228.30	16579.81	151503.05	3871.71	171410.52	161.98

**Table 6:** Statistics of predicted values of carbon density ARIMS model (Unit: gC/m^2^).

Year	Carbon density of cultivated land	Forest carbon density	Shrub carbon density	Grassland carbon density	Lake carbon density	Wetland carbon density
2025	513.44	567.87	469.28	273.56	205.13	229.52
2030	504.38	567.87	469.28	273.56	229.92	229.52

**IM estimation results:** The average value of the estimated results of the Grey Prediction model and the ARIMA model was used as the estimated carbon density and land use areas, and the average value of the land use transfer probability of the Sichuan mountainous areas from 2000 to 2020 was used as the transfer probability in 2025 and 2030. The impact of land use change on ecological carbon sequestration in Sichuan mountainous areas in 2025 and 2030 was estimated by incorporating them into the calculation equation (Equation 1–3) of the impact model. However, the Grey Prediction model and ARIMA prediction model are based on existing basic data to estimate future trends. They are based only on the data calculation in the state of the data itself, ignoring the impact of the environment on the data. Combined with the trend analysis of land use, carbon density, and total carbon sequestration data (Table 1, Figs 5–11), the average values of lake area, ice and snow area, wasteland area, wetland carbon density, and lake carbon density from 2000 to 2020 were used as the estimated data for 2025 and 2030. In summary, the results of land use data and carbon density estimation in Sichuan mountainous areas in 2025 and 2030 (Tables 7 and 8):

**Table 7:** Statistics of the average predicted area of land-use type in 2025 and 2030 (Unit: km², Ton, g/m²).

Year	Construction land	Cultivated land	Forest	Shrub	Grass land	Wetland	Lake	Ice and snow	Wasteland
2025	203.31	15343.19	142642.40	3464.18	160418.40	357.96	1271.14	3443.62	1355.81
2030	228.92	15157.39	143514.87	3390.87	159787.52	349.85	1271.14	3443.62	1355.81

**Table 8:** Statistics of predicted average carbon density of land-use type in 2025 and 2030 (Unit: km², Ton, g/m²).

Year	Carbon density of cultivated land	Forest carbon density	Shrub carbon density	Grassland carbon density	Lake carbon density	Wetland carbon density
2025	544.02	554.14	477.14	265.51	149.1	184.81
2030	558.65	556.86	494.88	267.01	149.1	184.81

The IM calculates the impact of land-use type changes on ecological carbon sequestration in 2025 and 2030: the transfer-in (TI) impact and transfer-out (TO) impact of nine land-use type changes on ecological carbon sequestration in the Sichuan mountainous areas (Table 9).

**Table 9:** Statistics of the impact of land-use type change on ecological carbon sequestration (Unit: km², Ton, g/m²).

Transfer types	Construction land	Cultivated land	Forest	Shrub		Grass land	Wasteland	Ice and snow	Lake	Waste land
2025ZR	-21411	121148	339850	69537		-152274	-310814	-38	-14678	-14240
2025ZC	1915	-140193	-86510	-86246		176012	62549	21515	5234	7892
Subtotal	-19496	-19045	253340	-16709		23738	-248265	21477	-9444	-6349
2030ZR	-21692	141865	313132	77103		-162945	-311312	-38	-14799	-14448
2030ZC	2156	-163057	-59662	-101852		186785	62898	21616	5556	7858
Subtotal	-19535	-21192	253470	-24750		23839	-248414	21578	-9243	-6590
Total impacts of different types of land use transfers in 2025					-20753.69					
Total impacts of different types of land use transfers in 2030					-30837.03					

**Fig 14:**
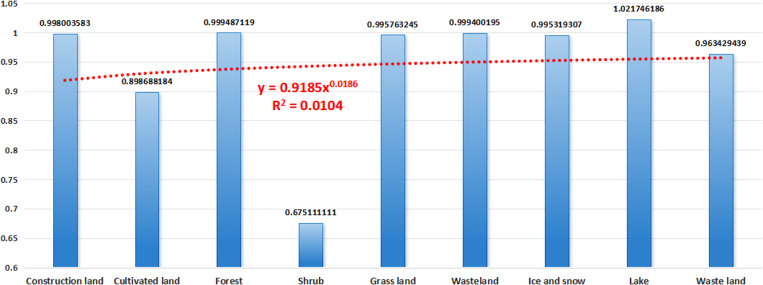
Comparison histogram of the impact of land use change on carbon sequestration in 2025 and 2030.

The changes in forest, grassland, ice, and snow are conducive to the improvement of ecological carbon sequestration capacity, while the changes in other land-use type are not conducive to the improvement of ecological carbon sequestration capacity. In 2025, the impact of land use change on ecological carbon sequestration capacity is -20754 tons, and the impact in 2030 is -30837 tons. At the same time, the impact of transfer between different land-use types on ecological carbon sequestration was different, but the impact of changes in the same land-use type at different periods on ecological carbon sequestration capacity was small. Comparing the impact of the same type of transfer in 2025 with that in 2030 (Fig 14), it is found that the impact of the same type of use change in 2025 accounts for 95% of the impact in 2030, and R^2^ is equal to 0.0104. The increase in the impact of future land-use change on ecological carbon sequestration capacity in Sichuan mountainous areas tends to be stable, and the impact will fluctuate around −31,156 tons (-30,837 * (1 + 0.0104)) in the future.

**Accuracy verification:** The improved CASA model improved the estimation accuracy of the CASA model, and its estimation results were considered close to the actual carbon sequestration amount. To verify the estimation accuracy of the IM, the IM was used to estimate the total amount of ecological carbon sequestration and the impact of land use change on ecological carbon sequestration in the study area in 2021 and 2022, and the improved CASA model was used to estimate the total amount of ecological carbon sequestration in 2021 and 2022. The two estimation results were compared to verify the estimation accuracy of IM. The equation for calculating the ecological carbon sequestration in 2021 and 2022 using IM is as follows:


$$GTZG=\sum n(S_{\left(i,t\right)}*\rho_{\left(i,t\right)})+GTYX_{t}$$ (5)

In the equation, GTZG is the total amount of ecological carbon sequestration in Sichuan mountainous areas, n is the number of land-use type in Sichuan mountainous areas, S_(i,t)_ is the estimated area of the i-th type of land-use type during the t period, *ρ*_(i,t)_ is the estimated carbon density of the i-th type of land-use type during the t period, and GTYX_t_ is the impact of land use change on ecological carbon sequestration. According to the land-use type areas and carbon density estimated by the IM, it is calculated that the total ecological carbon sequestration in 2021 without the impact of land use change is 121,335,540 tons, and the total ecological carbon sequestration in 2022 is 119,588,769 tons (Table 10).

**Table 10:** Statistics of total ecological carbon sequestration in 2021 and 2022 without considering the impact of land use change (Unit: 10,000 tons).

Year	Carbon sequestration in cultivated land	Carbon fixation in forest	Carbon fixation in Shrub	Carbon sequestration in grassland	Carbon sequestration in lake	Carbon sequestration in wetland	Total ecological carbon sequestration
2021	834.1070	7376.8004	163.6874	3833.9252	18.9521	6.0819	12133.5540
2022	833.5606	7396.2540	164.2869	3540.6517	18.9521	5.1716	11958.8769

The land use data and ecological carbon sequestration estimation data of Sichuan mountainous areas in 2021 and 2022 were imported into the IM, and the impacts of land use change on ecological carbon sequestration in 2021 and 2022 were calculated to be -82226 tons and -45892 tons, respectively (Table 11). Combined with the calculation results, the total amount of ecological carbon sequestration in 2021 was 12125.3314 million tons, and the total amount of ecological carbon sequestration in 2022 was 11954.2877 million tons.

**Table 11:** Statistics of the impact of land-use type changes on ecological carbon sequestration in 2021 and 2022 (Unit: tons).

Transfer types	Construction land	Cultivated land	Forest	Shrub		Grass land	Wasteland	Ice and snow	Lake	Wasteland
2021TI	-21374	113429	351906	265		-147450	-309065	-38	-14765	-14011
2021TO	1654	-132962	-100887	-74092		169303	62274	21435	5005	7148
Subtotal	-19720	-19533	251019	-73828		21853	-246791	21397	-9759	-6863
2022TI	-21385	119775	361451	8292		-169647	-295729	-38	-13755	-11908
2022 TO	1698	-139091	-98444	-84758		209267	59521	20633	3058	5168
Subtotal	-19687	-19316	263008	-76466		39620	-236208	20595	-10697	-6741
Total impacts of different types of land use transfers in 2021					-82226					
Total impacts of different types of land use transfers in 2022					-45892					

To verify the accuracy tiof the impact model, this study used the ecological carbon sequestration estimated by the IM to compare with the improved CASA model estimation and the ecological carbon sequestration calculated by MODIS data. The improved CASA model estimated that the total amount of ecological carbon sequestration in 2021 and 2022 will be 122.915 million tons and 1255.195 million tons, respectively, and the total amount of carbon sequestration calculated by MODIS data will be 123.7702 and 1258.101 million tons, respectively. In 2021, the estimated amount of IM accounted for 98.65% of the improved CASA model and 97.97% of the MODIS data, and the estimated IM in 2022 accounted for 95.24% of the improved CASA model and 95.19% of the MODIS data. Compared with the estimation results for 2021 and 2022, the estimation accuracy of the IM is higher, indirectly reflecting the accuracy of the IM in quantifying the impact of land use change on ecological carbon sequestration.

## Result and discussion

### Result

This study used the IM model to quantify the impact of land use change on ecological carbon sequestration, and the results showed the following:

1. The total ecological carbon sequestration in the Sichuan mountainous areas in 2025 and 2030 was 132 and 133 million tons, respectively (Table 12).
2. According to the model estimation, the impact of land use change on ecological carbon sequestration in the study area will be -20754 tons and -30837 tons in 2025 and 2030, respectively.
3. The impact of land use change on ecological carbon sequestration capacity tends to stabilize, with fluctuations of approximately −31156 tons.

**Table 12:** Estimation and statistics of total ecological carbon sequestration in 2025 and 2030 (Unit: 10,000 tons).

Year	Carbon sequestration in cultivated land	Carbon fixation in forest	Carbon fixation in Shrub	Carbon sequestration in grassland	Carbon sequestration in lake	Carbon sequestration in wetland	Impact of land use change	Total carbon sequestration
2025	834.7002	7904.3860	165.2899	4259.2689	6.6155	18.9527	-20754	13187.1378
2030	846.7676	7991.7691	167.8074	4266.4866	6.4656	18.9527	-30837	13295.1653

### Discuss

Advantages and disadvantages: Compared with other studies on the correlation between land use and carbon sequestration capacity, the biggest feature of this study is that it directly quantifies the impact of land-use type changes on carbon sequestration capacity by constructing an impact model and further analyzes the changing trends of their mutual influence. However, there are still some shortcomings in IM that need to be addressed. First, there is inevitably a certain degree of error in the analysis of the changing trends of land-use classification data and carbon sequestration data in the research area from 2000 to 2020, and the changing trends of both are key parameters required for IM. The accuracy of these key parameters has a significant impact on the IM estimation results. At present, the classification accuracy of land use data is not entirely accurate, and the estimation accuracy of carbon sequestration data is affected by many factors. Therefore, it is necessary to further improve the classification accuracy of land use and the accuracy of carbon sequestration estimation data to enhance the estimation accuracy of the IM. In addition, because of the large scope of the research area, it is difficult to consider the impact of local land policies, climate change, and other factors on the estimation results, and the influence of these factors on the estimation conclusions is difficult to assess. Finally, but not the endpoint, there are very few key parameters that affect the model, and there may still be some factors that are not fully included in the model.

Research direction: based on the above shortcomings, in future research, considering the variability of climate, human factors, policy factors, as well as the emergence of new methods and technologies, the IM should incorporate more influencing factors and continuously optimize calculation methods to improve the accuracy of impact model estimation.

## Conclusion

This study is based on land use data and ecological carbon sequestration data in mountainous areas of Sichuan Province from 2000 to 2020 and constructs an impact model from two aspects: the impact of land use transfer in and the impact of land use transfer out. The estimation model is used to estimate the impact of land use change on ecological carbon sequestration. The conclusions of this study are as follows.

1. The amount of ecological carbon sequestration in Sichuan mountainous areas shows a slow growth trend from 2000 to 2020, and the total amount of ecological carbon sequestration in 2025 and 2023 shows little difference (a difference of 0.01 million tons). This shows that the ecological carbon sequestration capacity of Sichuan mountainous areas is close to saturation, and the scientific management of the conversion between land-use type is conducive to the improvement of the ecological carbon sequestration function..
2. The impact of land-use change on ecological carbon sequestration tends to stabilize, and the current land-use change is not conducive to improving the ecological carbon sequestration capacity.
3. The model can effectively quantify the impact of land use on ecological carbon sequestration. The accuracy of the impact model is verified through comparison with relevant data from 2021 and 2022, which can intuitively demonstrate the impact of land-use change on ecological carbon sequestration, help policymakers grasp the impact of land-use change on ecological carbon sequestration capacity and its change trend, and have a clear understanding of the change law of land use change and ecological carbon sequestration capacity in the future.

## Supporting information

S1 File Paper dataset 1 Carbon sequestration data: Carbon sequestration data in Sichuan mountainous areas. (ZIP)

S2 File Paper dataset 2 Vector data within the research area: Vector data within the research area. (ZIP)

S3 File Paper dataset 3 Land use data (2000-2020): 2000-2002. (ZIP)

S4 File Paper dataset 3 Land use data (2000-2020): 2003-2005. (ZIP)

S5 File Paper dataset 3 Land use data (2000-2020): 2006-2008. (ZIP)

S6 File Paper dataset 3 Land use data (2000-2020): 2009-2011. (ZIP)

S7 File Paper dataset 3 Land use data (2000-2020): 2012-2014. (ZIP)

S8 File Paper dataset 3 Land use data (2000-2020): 2015-2017. (ZIP)

S9 File Map description. (DOCX)

S10 File Data availability statement. (DOCX)

S11 File Paper dataset 3 Land use data (2000-2020): 2018-2020. (ZIP)
