# Supplementary material for: A model of the impact of land use change on its carbon sequestration capacity
Source: PLoS One. 2025 May 29;20(5):e0323645. doi: 10.1371/journal.pone.0323645 (PMC12121811; doi:10.1371/journal.pone.0323645)
Supplement: S9 File — (DOCX) [file pone.0323645.s009.docx]

Data Availability statement

The minimum dataset involved in the paper mainly includes land use data in Sichuan mountainous areas, carbon sequestration data in Sichuan mountainous areas, and vector data of the boundaries of Sichuan mountainous areas, which can only be viewed and edited through ArcGIS software.

Experimental result cashback method and steps:

Firstly, using boundary vector data from Sichuan mountainous areas, extract land use data and carbon sequestration data to obtain land use data and carbon sequestration data in the study area.

Secondly, statistical analysis was conducted on land use data and carbon sequestration data in mountainous areas of Sichuan Province (land area data and carbon sequestration data from different years are listed in Table 1 of the paper), and the area change and carbon density change of different land use types were calculated to predict the development trend of area change and carbon density change (Figure 5-11 and Table 2 in the paper analyze the trend). The trend of area change and carbon density change are important factors for quantifying the impact of land use on ecological carbon sequestration.

Finally, these factors were incorporated into the calculation formula to estimate the impact of land use change on ecological carbon sequestration (Table 9 of the paper).

In order to reproduce the experimental results, I have uploaded the above three types of data to four databases (database details are as follows), but they have not been reviewed and published yet. For this reason, I also upload the above data as supporting information to you (**as an alternative method of uploading to the database**), hoping to use it to advance the progress of paper publication.

Repository Name 1: DANS Data Station Life Sciences

Dataset Name: Database on the Impact of Land Use Change on Carbon Sequestration

Data connection: https://doi.org/10.17026/LS/HFOBNA

Repository Name 2: FAIRsharing

Dataset Name: Database on the Impact of Land Use Change on Carbon Sequestration

(DILUCCS)

Data connection: <https://fairsharing.org/6403.>

Repository Name 3: DRYAD

Dataset Name: Database on the Impact of Land Use Change on Carbon Sequestration

Data connection: https://datadryad.org/dashboard.

Repository Name 4: Zenodo

Dataset Name: Dataset on the Impact of Land Use Change on Ecological Carbon Sequestration

Data connection: **10.5281/zenodo.15277235 (https://zenodo.org/uploads/15277235)**

**For the availability of data, the author promises that the above data can be shared and used for free and all relevant data are within the manuscript and its Supporting Information files.**
