# Supplementary material for: A model of the impact of land use change on its carbon sequestration capacity
Source: PLoS One. 2025 May 29;20(5):e0323645. doi: 10.1371/journal.pone.0323645 (PMC12121811; doi:10.1371/journal.pone.0323645)
Supplement: S10 File — (DOCX) [file pone.0323645.s010.docx]

Map Description

1. The acquisition method of Figures 1, 2, 4, and 12:

Figures 1, 2, 4, and 12 are not protected by copyright. Figures 1: The figure was cropped from DEM data and terrain undulation data in Sichuan Province based on the definition of mountainous areas, and the terrain undulation data of Sichuan Province is obtained through the calculation of Sichuan DEM data. The DEM data of Sichuan Province can be downloaded online (download link: [https://www.nesdc.org.cn/sdo/detail?id=62abfde27e281714dcb6038b).](https://www.nesdc.org.cn/sdo/detail?id=62abfde27e281714dcb6038b）。)Figures 2, 4, and 12 were obtained by cropping land use data and carbon sequestration data through Sichuan mountainous areas data, and the acquisition of land use data and carbon sequestration data were specifically introduced in the article.

Note: Data Usage Statement in DEM Data Sharing Platform:This platform owns copyright and other intellectual property rights to the data products or services it publishes or contributed by data contributors, as well as the related resources published on the platform, and are protected by law.Without the written permission of this platform, no organization or individual may copy, modify, transcribe, disseminate or sell any part of the above data products, services, information or materials in any way or for any reason.Anyone who infringes upon the copyright and other intellectual property rights of this platform will be held legally accountable by this platform. We hereby solemnly declare this!

This data usage instruction is for the data shared by the platform, "2000 Global 30m Vegetation Region SRTM DEM Correction Product," and not for other research data generated based on this dataset. And the sharing platform clearly expresses the sharing and referencing methods of the data:

Data sharing method

Public sharing: Download data directly after filling in the purpose

Data Usage Statement

To respect intellectual property rights and protect the rights and interests of data authors and data service providers, data users are requested to clearly label the data authors and sources in the research results (including project evaluation reports, acceptance reports, academic papers or graduation theses, etc.) generated based on this data, and indicate the references and data citations according to the [citation method]. At the same time, electronic versions of publicly available results should be sent to email addresses nesdc@igsnrr.ac.cn Please set the email subject to 'National Ecological Science Data Center Resource Sharing Service Published Results'.

Figures 1, 2, 4, and 12 of the paper are new datasets generated based on this dataset. The author has indicated the application and download link of this dataset in the article according to the requirements of the sharing platform. Therefore, the authors believes that Figures 1, 2, 4, and 12 do not infringe on the copyright of this dataset.

1. Map processing software: ArcGIS 10.8 was used in the paper to process Figures 1, 2, 4, and 12. The download link for this software is : https://soft.wxqilinz.cn/gis-jb61d/?plan=gisce&unit.
